# Clinical phenotype and genotype of children with GABA_A_ receptor α1 subunit gene-related epilepsy

**DOI:** 10.3389/fneur.2022.941054

**Published:** 2022-07-20

**Authors:** Linlin Zhang, Xinjie Liu

**Affiliations:** Department of Pediatrics, Qilu Hospital of Shandong University, Jinan, China

**Keywords:** GABRA1, epilepsy, West syndrome, Dravet syndrome, GEFS+

## Abstract

**Objective:**

This study aimed to summarize the clinical phenotype and genotype of children with epilepsy caused by GABRA1 gene variants.

**Methods:**

Eight epilepsy patients, who were admitted to Qilu Hospital of Shandong University from 2015 to 2021, were enrolled in the study. GABRA1 gene variants were detected by whole-exome sequencing. Epilepsy clinical manifestations, electroencephalography, neuroimaging characteristics and treatment methods were retrospectively analyzed.

**Results:**

Among the eight patients, four were males and four were females. Epilepsy onset age was between 3 and 8 months of age. Two patients had a family history of epilepsy. Six cases were *de* novo variants, and two were hereditary variants. Two children carried the same pathogenic variants, and five carried novel pathogenic variants that had not been reported internationally. The types of seizures were diverse, including focal seizures in five cases, generalized tonic-clonic seizures in five cases, and spasms in two cases. Electroencephalography of seven cases showed abnormal background rhythms, and six cases showed abnormal discharge during the interictal period. No obvious abnormalities were found on magnetic resonance imaging in five cases. All eight children had different degrees of developmental retardation.

**Conclusion:**

*De novo* pathogenic variants in GABRA1 are more common than inherited pathogenic variants, and most epilepsy symptoms begin in the first year of life, manifesting with a variety of seizure types and developmental delays. Conventional treatment usually involves one or more drugs; although drug treatment can control seizures in some cases, cognitive and developmental deficits often exist. The five newly discovered pathogenic variants enrich the GABRA1 gene pathogenic variant spectrum.

## Introduction

GABA_A_R (γ-aminobutyric acid type A receptor) is the primary inhibitory receptor of the central nervous system, mediating the fast inhibitory synapses of the nervous system (1). GABA_A_Rs are pentameric and are composed of different combinations of six α subunits, three β, γ or ρ subunits, and one ε, δ, θ, or π subunit (1). GABA (γ-aminobutyric acid) act in GABA_A_Rs to increase the membrane permeability of bicarbonate and chloride ions (2). In most mature neurons, postsynaptic GABA_A_R may be activated after high concentrations of GABA are released from presynaptic vesicles, causing a net inward flow of anions that hyperpolarize and inhibit postsynaptic potentials (2). Pathogenic variant of ion channels destroys the balance of excitatory and inhibitory neurotransmission, resulting in hyperexcitability and disinhibition in the brain (3). *In vitro* experiments show that many GABA_A_Rs that are mutated within the α subunit often exhibit reduced sensitivity to GABA and decreased current amplitudes, resulting in subsequent reductions in inhibitory inputs in neurons, thereby increasing seizure susceptibility *in vivo* (4).

Epilepsy is a highly heterogeneous disease caused by genetic and structural or metabolic disorders of the central nervous system, and more than five hundred genes have been identified as being involved in epilepsy (5). Genetic epilepsy is an epilepsy syndrome associated with gene pathogenic variants including duplications, deletions detected by genetic and sequencing techniques, as well as chromosomal abnormalities (e.g., ring 20) tested by chromosomal microarray analysis and karyotype (3). Genetic epilepsy syndromes affect 30–50% of epilepsy patients with differing severities (6). The GABA_A_R α1 subunit (GABRA1) gene encodes the α1 subunit of the chloride ion channel, and is a member of the GABA_A_R gene family (7). GABRA1 pathogenic variants may attenuate the inhibitory function of GABA *via* haploinsufficiency, causing a broad spectrum of epilepsy phenotypes (4). The more severe early onset epilepsy in spectrum of GABA_A_R variants is summarized as developmental and epileptic encephalopathies (DEEs), often pharmacoresistant epilepsies and lead to developmental delays, intellectual deficits, and further neuropsychiatric symptoms, such as autism, movement disorders or dysmorphisms (8). Generalized epilepsies related to severe, recurrent seizures and cognitive declines are termed DEEs and include Dravet syndrome, West syndrome, Lennox-Gastaut syndrome and Ohtahara syndrome (3). Recent studies have shown that DEEs are related to many different GABA_A_R genes variants, and “sporadic” cases are often caused by *de novo* variants (8). Within this study, GABRA1 gene pathogenic variants were characterized by using whole exome sequencing technology in eight children with epilepsy, and gene phenotypes were combined with clinical manifestations to develop a new direction for clinical diagnosis and treatment.

## Methods

### Patients

Eight patients with pediatric epilepsy who visited the Department of Pediatrics of Qilu Hospital, China were enrolled and medical records were retrospectively analyzed (four boys and four girls). All patients were examined and diagnosed using a combination of patients' illness history, previous history, family history, physical examinations, developmental evaluation, electroencephalography (EEG) monitoring, magnetic resonance imaging (MRI) and genetic sequencing. Seizure types and epilepsy syndromes were diagnosed and classified according to the guidelines of International League Against Epilepsy (ILAE) (2017 and 2022) (9, 10).

### Genetic analysis

Genomic DNA was extracted using a QIAamp Blood Midi Kit (QIAGEN, Valencia, CA). An Illumina NextSeq 500 sequencer (Illumina, San Diego, CA, USA) was used with 150 bp paired-end reads. After sequencing, the raw data were saved in FASTQ format. Quality control (QC) filters were applied to remove reads with low quality. Clean reads were assembled and spliced using the second-generation sequencing analysis platform provided by MyGenostics and the coverage and sequencing quality of the target region were evaluated. A flash analysis platform was used to analyze the pathogenicity of variation, and possible variation loci were determined. The pathogenicity of variation loci was analyzed according to the ACMG (American College of Medical Genetics and Genomics) genetic variation classification criteria and guidelines (11). The genome reference sequence was hg19. An ABI3730xl sequencer (Applied Biosystems, USA) was used for Sanger sequencing, and the Sanger sequencing results were compared to the capture sequencing results. More details on DNA library preparation, enrichment and sequencing of targeted genes, bioinformatics analysis, the whole genome CNV analysis, variants selected and software and database are listed on the Supplementary Material.

### Conservative and *in silico* analysis

A conservative analysis of the eight mutant amino acid sequences was performed using Clustal Omega. GABRA1 domains were identified based on data from the National Center for Biotechnology Information (NCBI) Conserved Domain Database. Multiple sequence alignments of GABRA1 were performed using the ClustalW program. Three-dimensional structural models of GABRA1 were predicted with the Swiss-model web tool. Protein structure images were generated using the PDB file and PyMOL. Hydrogen bonds within proteins were visualized using PyMOL to predict changes in mutant stability.

## Results

### GABRA1 gene pathogenic variant detection and gene analysis

**Figure 1:**
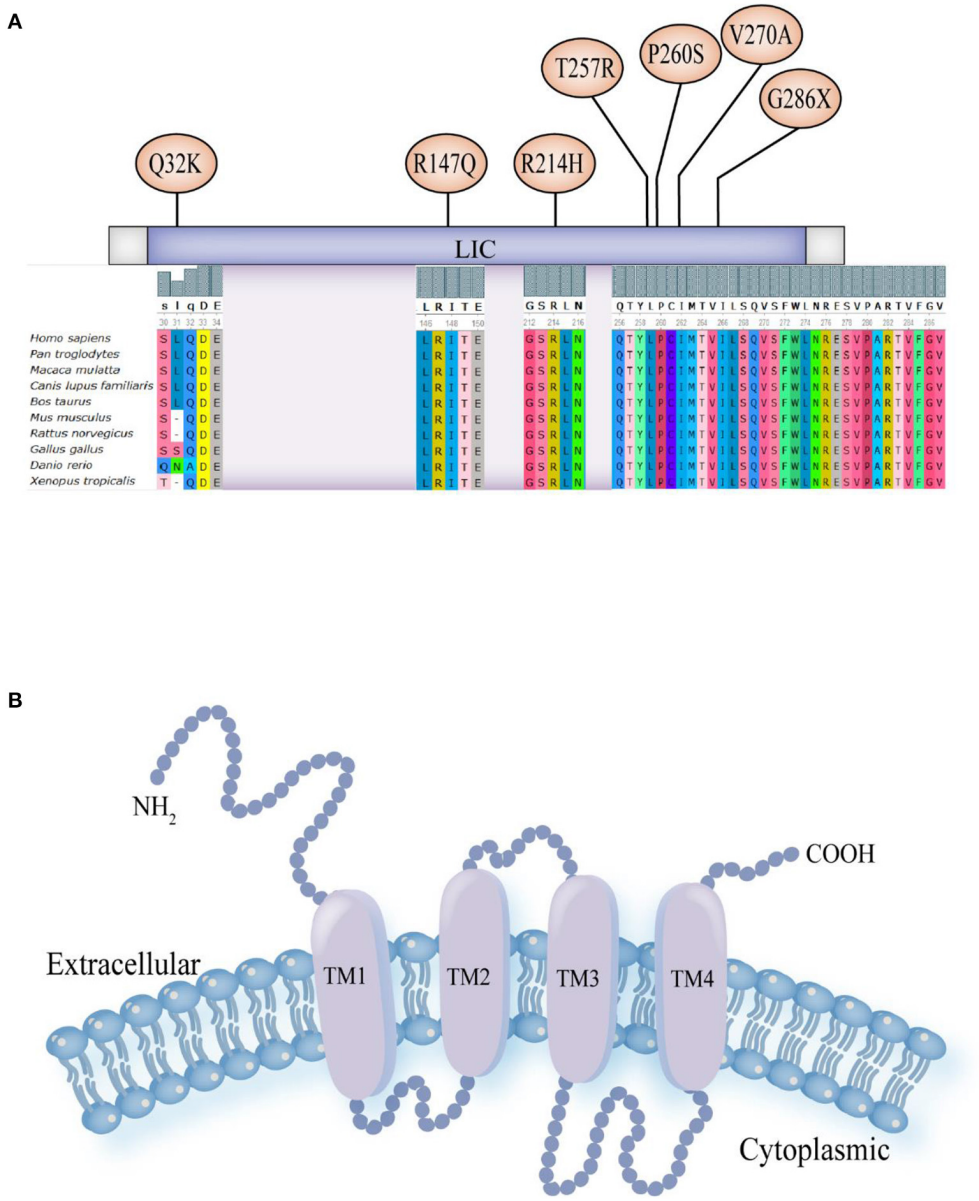
**(A)** Schematic diagram of pathogenic variant sites in nucleic acids and the results of multiple sequence alignment analysis of eight patients: p. Gln32Lys (Q32K), p. Arg147Gln (R147Q), p. Arg214His (R214H), p. Thr257Arg (T257R), p. Pro260Ser (P260S), p. Val270Ala (V270A) and p. Gly286X (G286X). **(B)** Schematic model for the protein structure of GABA_A_R subunits. The extracellular domain N-terminus, followed by the transmembrane domain containing four transmembrane segments (helices TM1-4). There is a small extracellular loop between TM2 and TM3, a larger intracellular loop between TM3 and TM4, and the TM2 helices form the ion pore.

In the eight patients with epilepsy, all GABRA1 gene variants were heterozygous. Seven of the GABRA1 gene variants were missense pathogenic variants, and one (case 5) was due to a nonsense pathogenic variant (Table 1). Two cases were inherited (case 2, c.94C>A, p. Q32K, maternal inheritance; case 5, c.856G>T, p. G286X, paternal inheritance). The remaining six cases were *de novo* pathogenic variants. Three cases carrying pathogenic variants were internationally reported variations, including two cases of p. R214H and one case of p. P260S. Five new variation sites that had not been reported internationally were identified, including p.T257R, p.Q32K, p.R147Q, p.V270A, and p. G286X (Figure 1A). The variable site of the GABRA1 gene pathogenic variant was highly conserved in different species according to multiple sequence alignment analysis.

**Table 1:** Summary of the variants in the GABRA1.

**Case**	**Gene/**	**Transcript**	**Exome**	**Nucleotide**	**Amino**	**Homozygous/**	**Sources of**	**ACMG**	**Diagnosis**
**code**	**location**			**changes**	**acid changes**	**heterozygous**	**variation**	**pathogenicity**	
1	chr5-161317970	NM_000806	9	c.770C>G	p. T257R	Het	*De novo*	LP	IESS
2	chr5-161281183	NM_000806	4	c.94C> A	p. Q32K	Het	Mother	VUS	Generalized onset
3	chr5-161300307	NM_000806	6	c.440G>A	p. R147Q	Het	*De novo*	LP	GEFS+
4	chr5-161318009	NM_000806	9	c.809T> C	p. V270A	Het	*De novo*	LP	FS
5	chr5-161318056	NM_000806	9	c.856G>T	p. G286X	Het	Father	LP	Generalized onset
6	chr5-161309645	NM_000806	8	c.641G>A	p. R214H	Het	*De novo*	P	DS
7	chr5-161309645	NM_001127648	6	c.641G> A	p. R214H	Het	*De novo*	P	FS
8	chr5-161317978	NM_001127648	7	c.778C> T	p. P260S	Het	*De novo*	P	IESS

*The genomic reference consortium build: GRCh37*.

*Het, heterozygous; LP, likely pathogenic; VUS, variant of uncertain significance; P, pathogenic; IESS, Infantile epileptic spasms syndrome; GEFS+, Genetic epilepsy with febrile seizures plus; FS, Febrile seizures; DS, Dravet syndrome*.

Three-dimensional structure predictions showed changes in the spatial structure of the GABRA1 protein after gene pathogenic variant (Supplementary Figure S1). In case 1 (c.770C>G, p. T257R), the Thr at position 257 was replaced by Arg, resulting in formation of an extra amino acid side chain (Supplementary Figure S1A). In case 3 (c.440G>A, p. R147Q), the Arg at position 147 was replaced by Glu, and the H-bonds and complex structure did not change (Supplementary Figure S1B). In case 4 (c.809T> C, p. V270A), the Val at position 270 was replaced by Ala, and the H-bonds and complex structure did not change (Supplementary Figure S1C). In case 5 (c.856G>T, p. G286X), a heterozygous G to T transition at position 856 in exome 9 encoded to a stop codon, resulting in a nonsense pathogenic variant and loss of gene function (Supplementary Figure S1D). Sanger verification confirmed that the four observed pathogenic variants were heterozygous. According to ACMG criteria, c.770C>G (p. T257R), c.440G>A (p. R147Q), and c.809T>C (p. V270A) were PS2+PM2+PP3 (likely pathogenic), and c.856G>T (p. G286X) was PVS1+PM2 (likely pathogenic).

### Clinical phenotype

**Figure 2:**
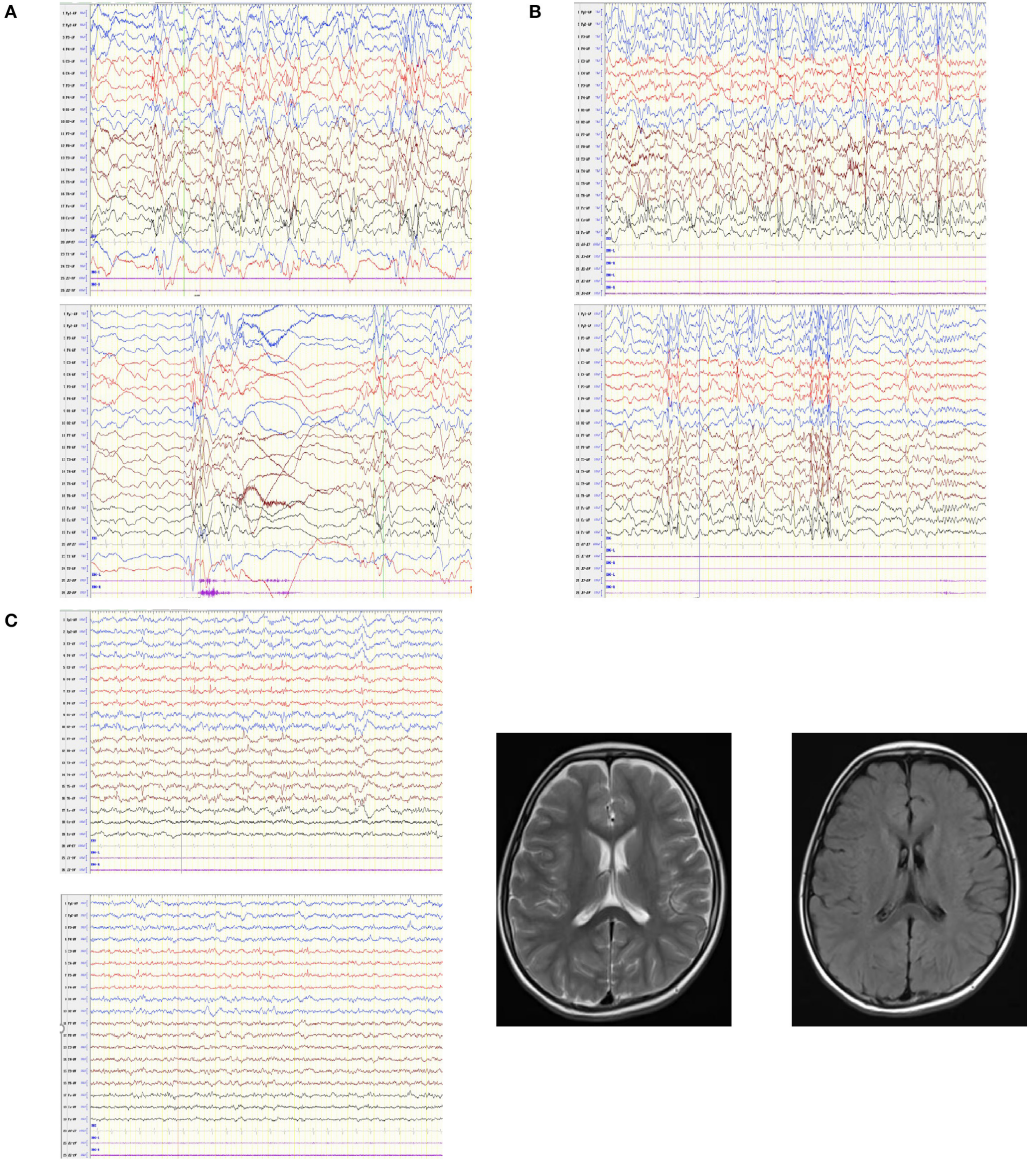
EEGs in patients with GABRA1 gene pathogenic variants and the MRI of one patient. **(A)** Case 1: hypsarrhythmia, multiple isolated and cluster or focal convulsive episodes. **(B)** Case 8: hypsarrhythmia, multifocal spike wave, spike slow wave, and poly-spike slow wave. **(C)** Case 4: slow background rhythm, spike wave and spike slow complex wave; patient's MRI was normal.

In all eight cases, the age of epilepsy onset was concentrated between three-eight months of age, with the exception of case 5. Of the eight patients, four had multiple seizure types. Five cases (cases 2, 4, 5, 7 and 8) had focal seizures, five cases (cases 3, 4, 6, 7 and 8) had generalized tonic-clonic seizures and two cases (cases 1 and 8) had spasms. Four cases (cases 3, 4, 6 and 7) have had heat sensitivity (Table 2). All eight children were monitored by video EEG one to seven times. Abnormal EEGs in seven children were all accompanied by abnormal background rhythms. Abnormal discharges in the interictal period were recorded in six cases, including three cases (cases 5, 7 and 8) of generalized discharges, two cases (cases 4 and 6) of focal discharges, and three cases (cases 1, 7 and 8) of multifocal discharges. Four patients had seizures during the detection period, two patients (cases 1 and 8) had spasms, one patient (case 3) had focal discharge, and one patient (case 5) had a generalized spike wave (GSW) in the posterior head that was induced by flash stimulation of the open eye. The craniocerebral MRI of five patients showed no obvious abnormalities (Figure 2C).

**Table 2:** Clinical features of patients with GABRA1 pathogenic variants.

**Case**	**Gender/**	**Age of**	**Family**	**Seizure**	**EEG**	**Brain**	**Diagnosis**	**Developmental**	**Drug**	**Drug**
**code**	**age**	**onset**	**history**	**types**		**MRI**		**delay**		**responsiveness**
1	F/2 y3 m	4 m	No	Spa.	Hypsarrhy., BS, Multi. FD	Normal	IESS	Severe	ACTH/VPA, TPM, NZS/PER, CLB	Not alleviated
2	F/5 y4 m	7 m	Mother	FoS (I)	Normal (7 m)	The left side ventricle is relatively wide compared to the other side	Generalized onset	Moderate	–	–
3	M/5 y7 m	8 m	Maternal grandmother	FS, GTCS	FSW	Normal	GEFS+	Moderate	LEV	Remission
4	F/8 y5 m	5 m	No	FS, GTCS, FoS	FSW	Normal	FS	Moderate	VPA, TPM, CZP	Remission
5	M/13 y	6 y	No	FoS (I)	GSW	Normal	Generalized onset	Moderate	OXC, TPM	Remission
6	M/9 y	8 m	No	FS, GTCS	FSW	Not performed	DS	Severe	LTG/VPA, TPM, CZP	Not alleviated
7	M/12 y	5 m	No	FS, GTCS, FoS	Multi. FD, GSW	Not performed	FS	Moderate	VPA, TPM	Remission
8	F/17 y	3 m	No	Spa., FoS, GTCS	Hypsarrhy., BS, Multi. FD, GPSW	Normal	IESS	Profound	ACTH/VPA, LEV, OXC, LTG/CLB, ZNS	Not alleviated

*F, female; M, male; y, year; m, month; IESS, Infantile epileptic spasms syndrome; GEFS+, Genetic epilepsy with febrile seizures plus; FS, Febrile seizures; DS, Dravet syndrome; Spa., Spasms; FS, Febrile seizure; FoS (I), focal seizures (impaired awareness); GTCS, generalized tonic-clonic seizures; FoS, focal seizures; Hypsarrhy., Hypsarrhythmia; BS, burst suppression; Multi. FD, multifocal discharges; FSW, focal spike wave; GSW, generalized spike-wave; GPSW, generalized polyspike-wave; ACTH, adrenocorticotropic hormone; VPA, valproic acid; TPM, topiramate; NZS, zonisamide; PER, perampanel; CLB, clobazam; LEV, levetiracetam; CZP, clonazepam; OXC, oxcarbazepine; LTG, lamotrigine*.

Some children enrolled in the study had a similar family history; the mother in case 2 had a history of seizures controlled by oral medication, and the grandmother in case 3 had a history of febrile seizures (FS). Some children are accompanied by other system diseases. Case 1 accompanied by sinus arrhythmia with occasional ventricular escape, case 3 has descended left testicle and case 7 was diagnosed as congenital laryngeal wheezing and mesenteric lymphadenitis. Patients 3, 4, 6 and 7 have convulsions with fever. Children with seizures may be accompanied by somatic symptoms. The onset age of case 5 was elder, accompanied by abdominal pain. At 7 and 9 years old, the seizure occurred again, including abdominal pain, headache, double vision and vomiting. At 10 years old, the headache was relieved, but paroxysmal abdominal pain still existed, and urinary incontinence occurred. In case 7, each fever convulsion was caused by upper respiratory tract infection. At 4–5 years of age, there was no fever convulsion, accompanied by vomiting and abdominal pain. Case 8 developed convulsions after vaccination at the age of 3 months, and remained in bed for a long time. At the age of 13, she suffered frequent seizures due to head injury. At the age of 17, she was admitted to Pediatric Intensive Care Unit for severe pneumonia and respiratory distress. All eight children showed language and motor development retardation.

### Treatment and prognosis

Two cases of West syndrome were treated with adrenocorticotropic hormone (ACTH), with undesirable outcomes. Case 1 was previously treated with topiramate (TPM), valproic acid (VPA), and zonisamide (NZS) but is currently treated with perampanel (PER) and clobazam (CLB). Despite the current treatment regimen, case 1 still had seizures. In case 8, oxcarbazepine (OXC), lamotrigine (LTG) and other drugs had been previously administered for treatment. The current treatment regimen for case 8 included levetiracetam (LEV), VPA, CLB and ZNS. Despite previous and current treatment, case 8 presented with severe cognitive and motor retardation. In case 3, LEV was the only drug administered for treatment, although the treatment dose had been adjusted several times. Case 3 symptoms were well-controlled but accompanied by cognitive deficits and inattention. Four patients who took either TPM, VPA, OXC, or clonazepam (CZP) or a combination of these drugs exhibited differing degrees of mental or motor retardation.

The last follow-up age of the eight enrolled patients ranged from 2 years and 3 months to 17 years old. Four patients were in remission at 2 years and 5 months to 10 years old and remained in remission for 3–8 years at the time of this study. One patient had had a seizure following a 3-year period of remission. The seizure was reportedly different from the patient's previous seizures. Three epilepsy cases were not alleviated with treatment; one epilepsy case was lost to follow up. Among the four patients in remission, one patient received LEV monotherapy, one patient was treated with VPA and TPM, one patient received a combination OXC and TPM treatment, and one patient received a combination VPA, TPM, and CZP treatment. The three children who continued to have seizures were treated with at least three to four anti-seizure medications (ASMs).

## Discussion

### GABRA1 gene pathogenic variant

The GABRA1 gene is located on chromosome 5q34 and contains ten exomes and encodes four hundred and seventy-four amino acids (7). All GABA_A_R subunits have a similar protein structure: an extracellular domain N-terminus, followed by the transmembrane domain, which includes four transmembrane segments (helices TM1-4) with a small extracellular loop between TM2 and TM3 and a larger intracellular loop between TM3 and TM4 and TM2 helices that form the ion pore (Figure 1B) (3, 12). Research has shown that gene pathogenic variants that are present in epilepsy patients are clustered at the N-terminus, TM2, and TM3 (8). Epileptic disorders caused by pathogenic variants in GABRA1 cause a wide range of clinical symptoms, including mild generalized epilepsies and severe infantile DEEs (13).

The current study included patients with epilepsy types of varying severity, including GEFS+, FS, and more severe DEEs, such as West syndrome and Dravet syndrome. In this study, all eight children had pathogenic variants in the GABRA1 gene and seven pathogenic variants of GABRA1 were identified: c.770C>G (p. T257R), c.94C> A (p. Q32K), c.440G>A (p. R147Q), c.809T> C (p. V270A), c.856G>T (p. G286X), c.778C> T (p. P260S, and two c.641G>A (p. R214H) pathogenic variants. Among these pathogenic variants, p. T257R, p. Q32K, p. R147Q, p. V270A, p. G286X were novel pathogenic variants found in five separate patients. Sanger sequencing verification showed that these pathogenic variants were heterozygous. P. Q32K and p. G286X were the only inherited pathogenic variants. However, the parents who carried these relevant pathogenic variants did not develop the disease, which may be due to incomplete penetrance. In addition, p. G286X was identified as a nonsense pathogenic variant, whereas the other pathogenic variants were missense pathogenic variants. Nonsense pathogenic variants can lead to premature termination of amino acid translation, and is generally presumed to be a likely pathogenic variant.

In our study, c.770C>G (p. T257R) and c.778C>T (p. P260S) pathogenic variants were noted in two patients diagnosed with West syndrome and a c.641G>A (p. R214H) pathogenic variant was noted in a patient diagnosed with Dravet syndrome. Notably, West syndrome and Dravet syndrome are types of early-onset epileptic encephalopathies (EOEEs). Hirofumi Kodera et al. identified five pathogenic variants in six patients. Of these six patients, four were diagnosed with either West syndrome with concomitant *de novo* pathogenic variants in the TM1 domain of GABRA1 (p. P260 L, p. M263T, and p. M263I) or with Ohtahara syndrome (p. P260 L, convert to West syndrome) (14). We found that the GABRA1 gene pathogenic variant in West syndrome was similar in location but showed changes in different bases of the same amino acid, resulting in different amino acids being encoded (p. P260S and p. P260 L, p. M263T, and p. M263I). There were also patients with the same pathogenic variant at the same site with different clinical manifestations, p. P260 L (Ohtahara syndrome, West syndrome) and p. R214H (Dravet syndrome, FS) in cases 6 and 7.

### GABRA1-related epilepsy clinical phenotype

DEEs are a heterogenous group of severe epilepsy disorders that display early seizure onset coupled with behavior and cognitive changes related to epilepsy activities (15). Hernandez et al. have shown that patients with a pathogenic *de novo* pathogenic variant of GABRA1 have a broad phenotypic range of severe EOEEs, such as West syndrome, Dravet syndrome, Ohtahara syndrome and epilepsy with myoclonic atonic seizures (formerly known as Doose syndrome) (16).

West syndrome, also known as epileptic spasms or infantile spasms which belong to infantile epileptic spasms syndrome (IESS), is often characterized by infantile spasm onset during 3–24 months of age, interictal hypsarrhythmia on EEG and developmental delay in 80–90% of patients (17). Case 1 (c.770C>G, p. T257R) and case 8 (c.778C>T, p. P260S) developed a series of nodding and hugging movements and heterphoria at the age of 3–4 months, accompanied by developmental delay, hypsarrhythmia on interictal EEG, and a normal brain MRI. Similar to the current findings, the previous study also noted that the GABRA1 pathogenic variant (c.778C>T, p. P260S) was associated with epileptic spasms with hypsarrhythmia on EEG and severe retardation in motor and cognitive development (6). The patient also showed a thin corpus callosum on MRI and did not respond well to ASMs; however, the combination of MgSO4 and ACTH reduced the patient's epileptic spasms (16). In addition, a *de novo* variant in GABRA1 (c.789G>A, p. M263I) has been observed. This variant is likely pathogenic and was associated with a distinct phenotype of West syndrome in a 9-month-old infant. The patient's symptoms were improved with TPM and steroid treatment (18). In contrast to this previous report, early intervention with ACTH in the current study did not result in an ideal treatment outcome. Thus, other drugs had to be used for symptom control. Despite the early drug intervention for seizure control, both cases presented with uncontrollable cognitive and behavioral regression. In combination with previous studies, we found that the West syndrome phenotype of the GABRA1 pathogenicity variants focuses on the amino acid changes at position 257–263. Although there is the same phenotype of the disease, there are differences in the severity of the disease and sensitivity to drug treatment due to the presence of different amino acid pathogenicity variants. Therefore, early identification of pathogenicity variants sites can help us make a correct judgment on disease and drug application. These observations also suggest some degree of genotype–phenotype correlation.

Case 6 (C.641G> A, P. R214H) was diagnosed with Dravet syndrome, also known as “severe myoclonic epilepsy of infancy”, which is a rare and catastrophic DEE (19). The seizures of Dravet syndrome typically begin in the first year of life (with an average age of onset being 6 months old) with prolonged generalized clonic or hemiclonic epileptic seizure onset, often triggered by fever (20). Multiple seizure types, including myoclonic seizures, focal dyscognitive seizures and atypical absence seizures, develop at ~2 to 5 years of age (21). Epilepsies are usually refractory to ASMs, and cognitive, behavioral, and motor impairments become apparent starting at the second year of life (22). In previous research, targeted resequencing was performed in sixty-seven patients with Dravet syndrome of typical disease progression and three pathogenic variants of GABRA1 were detected in four patients (c.751G> A, p.G251S ^*^1; c.335G> A, p.R112Q^*^2; c.917A> C, p.K306T^*^1) (23). Maria et al. identified a novel GABRA1 gene pathogenic variant (c.226A> C, p. S76R) in a fifteen-year-old boy with some atypical features of Dravet syndrome. Although his phenotype combined some of the key features of Dravet syndrome, the patient developed generalized tonic seizures followed by pyrexia at 13 months of age. All of the patient's EEGs showed slow background rhythms without epileptiform activity. The patient's seizures remained well-controlled during treatment with VPA, TPM, and CLB (19). Interestingly, we found that the pathogenic variation site of Dravet Syndrome is widely dispersed, ranging from position 76, 112, 214, 251–306. Therefore, pathogenic variants in the GABRA1 gene may present with both typical and atypical Dravet syndrome phenotypes.

### Drug resistance and drug response

Studies have shown that a lack of GABA may contribute to epilepsy and mood disorders. Many ASMs enhance GABAergic functions, including positive allosteric modulators of GABA_A_Rs and blockers of GABA uptake and degradation (24). Sodium valproate, a common anticonvulsant agent, is a structural analog of GABA that enhances GABA activity. Due to its GABAergic effects, valproate is used to treat mood disorders (25). In a study including fifteen children with a variant of the GABRA1 gene, seven were treated with ASMs and remained seizure free, including two patients with juvenile myoclonic epilepsy and one patient with GEFS+ treated with VAP monotherapy. LEV monotherapy was used in one case of idiopathic generalized epilepsy, one case of a Dravet-like phenotype, and one case with unspecified epilepsy. One patient with mild DEE was treated with the combination therapy of VAP plus LEV (4). In the present study, five patients were treated with VPA. Two patients with FS were seizure free and were treated with VPA combined with TPM or CZP. The other three patients with West syndrome and Dravet syndrome were treated with VPA combined with multiple drugs but still presented with seizures. There are four patients in remission in our study, including two patients with FS mentioned above, one patient with GEFS+ received LEV monotherapy and one patient received a combination OXC and TPM treatment.

### New pathogenic variants

The five newly discovered pathogenic variants which enrich the GABRA1 gene pathogenic variant spectrum include case 1 with West syndrome, case 3 with GEFS+, case 4 with FS, case 2 and 5 with uncertain epilepsy. Previous studies have shown that GABRG2 pathogenic variants encoding the γ2 subunit commonly occur in patients with simple FS and GEFS+(3), whereas GABRA1 pathogenic variants encoding the α1 subunit commonly occur in individuals with juvenile myoclonic epilepsy and childhood absence epilepsy (26). However, a cohort in the phenotypic spectrum of GABRA1 showed a wide range of epilepsy subtypes spanning from benign forms to moderately severe phenotypes and severe DEEs, with 6% of patients exhibiting a GEFS+ phenotype (4).

In the present study, case 3 (c.440G>A, p. R147Q), 4(c.809T> C, p. V270A) and 7(c.641G> A, p. R214H) had had more than two complex febrile convulsions within the first year of life, as characterized by seizures accompanied by fever, binocular squint, and limb rigidity. With increasing age, other forms of attack developed without fever, including absence attacks and physical symptoms. Although only case 3 had a family history of FS, their clinical manifestations were very similar, and the subsequent occurrence of GEFS+ in the families of case 4 and case 7 could not be ruled out. The patient with GEFS+ (c.220G> A, p. V74I) were seizure-free on VPA monotherapy in another study (4), and case 3 was also seizure-free on LEV monotherapy in the present study. Although both cases responded well to drug therapy, the patients inevitably displayed language and cognitive deficits. Case 5 (c.856G>T, p. G286X) was paternal inheritance and had generalized onset with GSW induced by flash stimulation. Previous study had shown that pathogenic variants in the GABRA1 gene should be considered in patients with DEE, especially those with infantile-onset, prominent tonic-clonic and myoclonic seizures, and GSWs and a photoparoxysmal response on the EEG (4). So we cannot ignore that genetic background and modifying genes might contribute to phenotypic heterogeneity.

### MRI and EEG findings

MRI inspection for the patient was to observe the abnormalities visible on structural neuroimaging, thus to judge whether the symptoms of seizure caused by a structural etiology (9). The MRI of the case 2 showed the left side ventricle is relatively wide compared to the other side, considering the developmental change. Combined with her mother also had symptoms of epilepsy, may provide further diagnosis of the cause of the disease. The craniocerebral MRI of five patients in our study showed no obvious abnormalities. Previous research has shown that if the result of the neuroimaging examination is normal, the patients usually have no or little cognitive impairment or neurodevelopmental comorbidities (27). However, in our clinical study, children with normal MRI results presented with varying degrees of language and behavior disorders. In recent guidelines of ILAE, MRI is not required for diagnosis of most epilepsies but is highly recommended to exclude other causes, and sometimes required for diagnosis to exclude a causal lesion (10). Combined with our research, MRI may be conducive to discovering certain structural etiology, but whether it is related to cognitive impairment, it also needs further research.

All abnormal EEGs in seven children were all accompanied by abnormal background rhythms. The EEG pattern of case 1 and 8 were similar, which was characterized by hypsarrhythmia, multiple single or cluster partial seizures, and outburst - suppression pattern (Figures 2A,B). However, in the diagnostic criteria for IESS, an ictal EEG is not required for diagnosis provided the interictal study shows hypsarrhythmia or epileptiform abnormalities or developmental delay (10). This shows that although characteristic abnormal EEG is helpful for the diagnosis of specific types of epilepsy, normal EEG can only indicate that abnormal discharge is not detected, and cannot exclude the possibility of epileptic seizures.

## Conclusion

Similar to the study of Ying Yang et al., *de novo* variation of GABRA1 was more common than inherited pathogenic variants, and most epilepsy caused by GABRA1 started within the first year of life with various seizure types and developmental delays (7). Cognitive and behavioral comorbidities, including intellectual disability, learning disorders, autism spectrum disorder, and attention deficit–hyperactivity disorder, are more common and severe in refractory epilepsy (17). Cognitive impairment or neurodevelopmental comorbidities are not precisely related to abnormal neuroimaging results but may be closely related to the type of epilepsy, the effect of drug treatment, and family or social factors.

In summary, *de novo* pathogenic variants in GABRA1 are more common than inherited pathogenic variants, and most epilepsy symptoms caused by GABRA1 begin in the first year of life. These patients present with a variety of seizure types, including GEFS+, FS, and more severe DEEs, such as West syndrome and Dravet syndrome, as well as with developmental delays. Each patient also showed varying degrees of EEG abnormalities and mostly normal MRIs. Conventional treatment usually involves one or more drugs; although medication can control seizures or reduce their frequency in some cases, cognitive and developmental deficits often persist. Due to the small number of cases in this study, the relationship between the clinical phenotype and genotype of GABRA1 gene pathogenic variants should continue to be explored.

## Data availability statement

The datasets presented in this article are not readily available due to government restrictions. Queries in regards to the data should be directed to the corresponding author(s).

## Author contributions

XL contributed to the conception of the work and revised it critically for important intellectual content. LZ acquired, analyzed the data, and drafted the work. Both authors approved publication of the content.

## Funding

This work was funded by grants from the Clinical research project of Shandong University (Key Project for Critical and Critical Care) in 2021 and the National Natural Science Foundation of China (82171352).

## Conflict of interest

The authors declare that the research was conducted in the absence of any commercial or financial relationships that could be construed as a potential conflict of interest.

## Publisher's note

All claims expressed in this article are solely those of the authors and do not necessarily represent those of their affiliated organizations, or those of the publisher, the editors and the reviewers. Any product that may be evaluated in this article, or claim that may be made by its manufacturer, is not guaranteed or endorsed by the publisher.
