# Supplementary material for: Clinical phenotype and genotype of children with GABAA receptor α1 subunit gene-related epilepsy
Source: Front Neurol. 2022 Jul 20;13:941054. doi: 10.3389/fneur.2022.941054 (PMC9350551; doi:10.3389/fneur.2022.941054)
Supplement: Supplementary file 1 [file Data_Sheet_1.docx]

***Supplementary Material***

**DNA Library Preparation**

Genomic DNA of 1 - 3 μg was fragmented to an average size of 150 bp using a S220 Focused-ultrasonicator (Covaris, Massachusetts, USA). A DNA Sample Prep Reagent Set (MyGenostics, Beijing, China) was used for the preparation of standard Illumina libraries, including end repair, adapter ligation, and PCR amplification, which would be further sequenced by DNBSEQ (DNBSEQ-T7).

**Enrichment and Sequencing of Targeted Genes**

The amplified DNA was captured using GenCap XXX capture kit (MyGenostics Inc, Beijing, China). The gene panel for XXX disease were downloaded from XXX database and summarized in Table 1. The biotinylated 100 bp capture probes were designed to tile along the coding exons plus 50 bp flanking regions of all the genes. The capture experiment was conducted according to manufacturer’s protocol. Briefly, DNA library of 500ng was mixed with Buffer BL and GenCap gene panel probes (MyGenostics Inc), firstly. The mixture was heated at 95°C for 5min, and then 65°C for 5 min on a PCR machine. After that, 19 μl of the 65°C prewarmed Buffer HY (MyGenostics, MD, USA) was added into the mixture, and this mixture was held at 65°C with PCR lid heat on for 16-24 hours for hybridization. 50 μl of MyOne beads (Life Technology) was washed using 50 μl of 1X binding buffer for 3 times, and then they were resuspended in 50 μl of 1X binding buffer. The hybrid mixture. Then, beads were washed with WB1 buffer at room temperature for 15 minutes once, and WB3 buffer at 65°C for 10 minutes three times. The bounded DNA was eluted with buffer, and amplified for 13 cycles using the following program: 95°C for 4min s (1 cycle); 98°C for 30 s, 65°C for 30 s, 72°C for 30 s (13 cycles); 72°C for 5 min (1 cycle). The PCR product was purified using SPRI beads (Beckman Coulter) according to manufacturer’s protocol. The enrichment libraries were sequenced on Illumina HiSeq X ten sequencer for paired-reading of 150 bp.

**Bioinformatics analysis**

After sequencing, the raw data were saved as a FASTQ format. Both Illumina sequencing adapters and low quality reads (< 80 bp) were filtered by cutadaptor software (<http://code.google.com/p/cutadapt/>). The clean reads were mapped to the UCSC hg19 human reference genome using the parameter BWA of Sentieon software (<https://www.sentieon.com/>).The duplicated reads were removed using the parameter driver of Sentieon software, and the parameter driver is used to correct the base, so that the quality value of the base in the reads of the final output BAM file can be closer to the real probability of mismatch with the reference genome, and the mapped reads were used for the detection of variation. The variants of SNP and InDel were detected by the parameter driver of Sentieon software. Then, the data would be transformed to VCF format. Variants were further annotated by ANNOVAR software (<http://annovar.openbioinformatics.org/en/latest/>), and associated with multiple databases, such as, 1000 genome, ESP6500, dbSNP, EXAC, Inhouse (MyGenostics), HGMD, and also predicted by SIFT, PolyPhen-2, MutationTaster, GERP++.

**The whole genome CNV analysis**

The whole genome Copy Number Variation (CNV) analysis obtains FASTQ format based on the above methods, and the sequencing adapters and low quanlity reads were filtered by cutadaptor software (<http://code.google.com/p/cutadapt/>). The clean reads were mapped to the UCSC hg19 human reference genome using the parameter BWA of Sentieon software (<https://www.sentieon.com/>). Then, CNVkit (<https://cnvkit.readthedocs.io/en/stable/>) software was used to obtain copy number variation information.

**Variants Selected**

In this study, four steps were used to select the potential pathogenic variants in downstream analysis: (i) Pathogenic variant reads should be more than 5, and pathogenic variant ration should be no less than 30%; (ii) The pathogenic variants should be removed, when the frequency of muta was more than 5% in 1000g, ESP6500, and Inhouse database; (iii) The pathogenic variants should be dropped, if they were in InNormal database (MyGenostics); (iV) The synonymous pathogenic variants should be removed, when they were not in the HGMD database. After that, the rest pathogenic variants should be the potential pathogenic pathogenic variants for further analysis.

**Software and Database**

**Sentieon:** <https://www.sentieon.com/>

**CNVkit:** <https://cnvkit.readthedocs.io/en/stable/>

**ANNOVAR：**<http://annovar.openbioinformatics.org/en/latest/>

Wang K, Li M, Hakonarson H. ANNOVAR: functional annotation of genetic variants from high-throughputsequencing data. Nucleic Acids Res. 2010; 38: e164.

**1000 genome：**<http://www.1000genomes.or/>

**EVS**：<http://evs.gs.washington.edu/EVS>

**dbSNP**：<http://www.ncbi.nlm.nih.gov/projects/SNP/>

**EXAC**：<http://exac.broadinstitute.org/>

**HGMD**：<http://www.biobase-international.com/product/hgmd>

**SIFT**：<http://sift.jcvi.org/>

**PolyPhen-2**：<http://genetics.bwh.harvard.edu/pph2/>

**MutationTaster**：<http://www.mutationtaster.org/>

**GERP++**：<http://mendel.stanford.edu/SidowLab/downloads/gerp/index.html>

**SPIDEX**：<http://www.deepgenomics.com/spidex>

Sentieon software was used form Oct,2020.


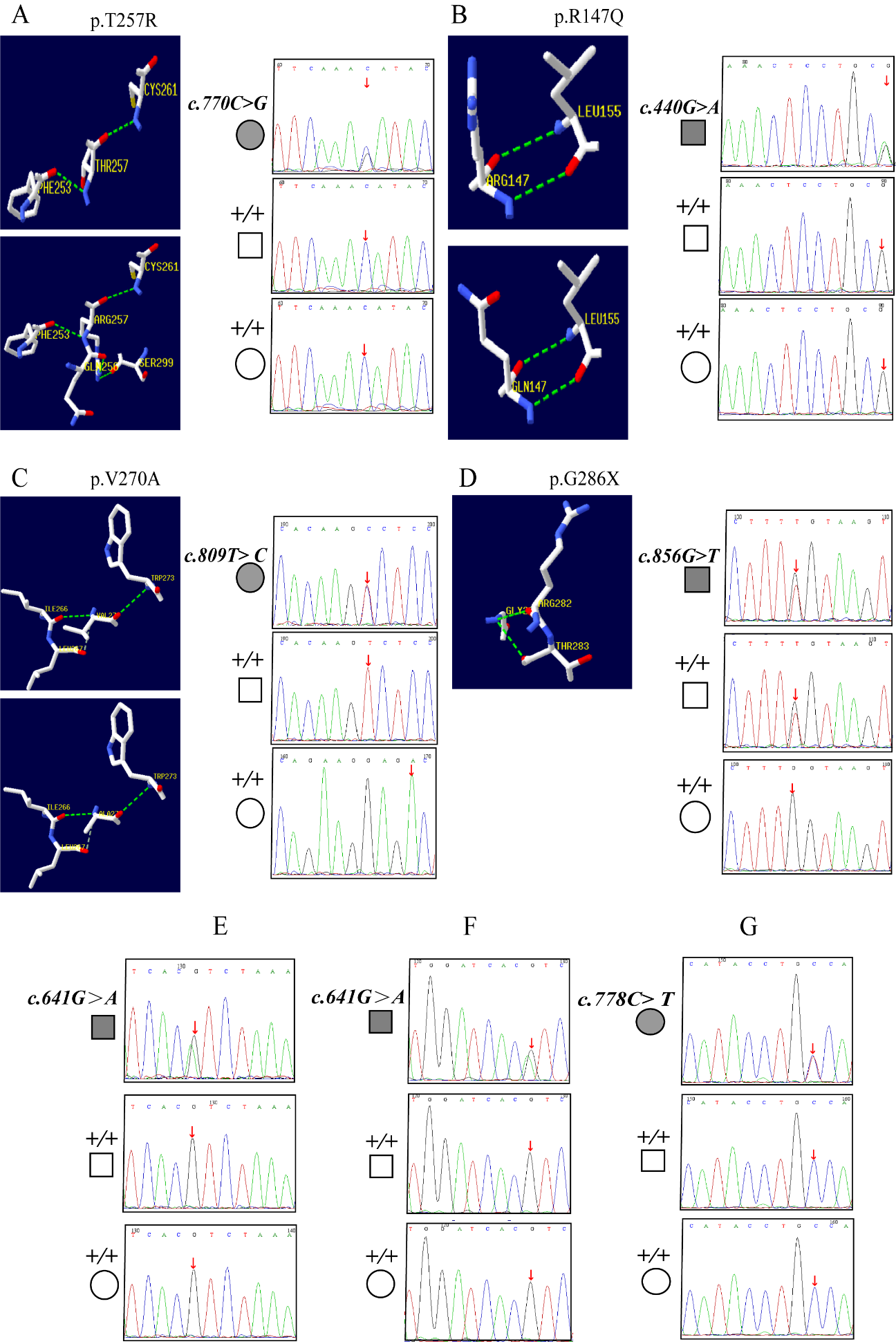


**Supplementary Figure 1.** The three-dimensional structure diagram of GABRA1 gene pathogenic variant sites and family gene analysis. Amino acid pathogenic varian sites are shown. Green dashed lines represent hydrogen bonds. (A-D) Pathogenic variant sites (cases 1, 3, 4, 5) that have not been reported worldwide, and (D) the nonsense pathogenic variant of case 5 was inherited from his father. (E-G) Previously reported pathogenic variant sites (cases 6, 7, 8) are shown. Genetic test results of the children and their parents showed missense pathogenic variants.
